# A Conserved Streptococcal Virulence Regulator Controls the Expression of a Distinct Class of M-Like Proteins

**DOI:** 10.1128/mBio.02500-19

**Published:** 2019-10-22

**Authors:** Jonathan D. D’Gama, Zhe Ma, Hailong Zhang, Xu Liu, Hongjie Fan, Ellen Ruth A. Morris, Noah D. Cohen, Colette Cywes-Bentley, Gerald B. Pier, Matthew K. Waldor

**Affiliations:** a Department of Microbiology, Harvard Medical School, Boston, Massachusetts, USA; b Division of Infectious Diseases, Brigham and Women’s Hospital, Boston, Massachusetts, USA; c MOE Joint International Research Laboratory of Animal Health and Food Safety, College of Veterinary Medicine, Nanjing Agricultural University, Nanjing, Jiangsu, China; d Ministry of Agriculture Key Laboratory of Animal Bacteriology, Nanjing, Jiangsu, China; e Jiangsu Co-Innovation Center for Prevention and Control of Important Animal Infectious Diseases and Zoonoses, Yangzhou, Jiangsu, China; f Department of Large Animal Clinical Sciences, College of Veterinary Medicine and Biomedical Sciences, College Station, Texas, USA; g Howard Hughes Medical Institute, Boston, Massachusetts, USA; Harvard Medical School

**Keywords:** M protein, *Streptococcus equi*, *Streptococcus zooepidemicus*, whole-genome sequencing, group A streptococcus, streptococcal pathogenesis, virulence regulation

## Abstract

Streptococcus equi subspecies *zooepidemicus* (SEZ) are group C streptococci that are important pathogens of economically valuable animals such as horses and pigs. Here, we found that many SEZ isolates bind to a monoclonal antibody that recognizes poly-*N-*acetylglucosamine (PNAG), a polymer that is found as a surface capsule-like structure on diverse microbes. A fluorescence-activated cell sorting-based transposon insertion sequencing (Tn-seq) screen, coupled with whole-genome sequencing, was used to search for genes for PNAG biosynthesis. Surprisingly, mutations in a gene encoding an M-like protein, *szM*, and the adjacent transcription factor, designated *sezV*, rendered strains PNAG negative. SezV was required for *szM* expression and transcriptome analysis showed that SezV has a small regulon. SEZ strains with inactivating mutations in either *sezV* or *szM* were highly attenuated in a mouse model of infection. Comparative genomic analyses revealed that linked *sezV* and *szM* homologues are present in all SEZ, S. equi subspecies *equi* (SEE), and M18 group A streptococcal (GAS) genomes in the database, but not in other streptococci. The antibody to PNAG bound to a wide range of SEZ, SEE, and M18 GAS strains. Immunochemical studies suggest that the SzM protein may be decorated with a PNAG-like oligosaccharide although an intact oligosaccharide substituent could not be isolated. Collectively, our findings suggest that the *szM* and *sezV* loci define a subtype of virulent streptococci and that an antibody to PNAG may have therapeutic applications in animal and human diseases caused by streptococci bearing SzM-like proteins.

## Abstract (precis)

M proteins are surface-anchored virulence factors in group A streptococci, human pathogens. Here, we identified an M-like protein, SzM, and its positive regulator, SezV, in Streptococcus equi subspecies *zooepidemicus* (SEZ), an important group of pathogens for domesticated animals, including horses and pigs. SzM and SezV homologues were found in the genomes of all SEZ and S. equi subspecies *equi* and M18 group A streptococcal strains analyzed but not in other streptococci. Mutant SEZ strains lacking either *sezV* or *szM* were highly attenuated in a mouse model of infection. Collectively, our findings suggest that SezV-related regulators and the linked SzM family of M-like proteins define a new subset of virulent streptococci.

## INTRODUCTION

Streptococcus equi subspecies *zooepidemicus* (SEZ) is a Lancefield group C streptococcus (GCS) that is a constituent of the normal upper respiratory tract flora of domesticated animals, such as horses (1). SEZ is also an opportunistic pathogen, and infections in a wide range of animals, including pigs, cows, dogs, and horses, have been reported (1, 2). Epizootic outbreaks in livestock (e.g., swine in China) can be widespread and severe, resulting in significant economic loss (3, 4). Infection of horses with another S. equi biovar, S. equi subspecies *equi* (SEE), causes the severe, contagious respiratory infection known as strangles (5). SEZ infection of humans, who typically acquire the pathogen through contact with infected animals or contaminated milk or cheese, typically causes meningitis and can be fatal (6, 7). Several virulence factors and protective antigens have been identified in SEZ/SEE, including two M-like proteins, referred to as SzM/SeM and SzP/SzPSe (8–10).

The M/M-like protein family is a class of surface-associated streptococcal proteins that includes M protein, a classical virulence factor of group A streptococcus (GAS; S. pyogenes) (11). Proteins in the group are anchored to the cell wall, share several features (including a primarily alpha-helical/coiled-coil structure, a polar cytoplasmic C-terminal tail, and an extracellular distal N terminus), and function in modulating the host immune response to the pathogen. The diverse immunomodulatory functions of M/M-like proteins include but are not limited to fibrinogen binding, inhibition of complement activation, antiphagocytic activity, and binding to Fc portions of immunoglobulins (11, 12). M/M-like proteins are typically key streptococcal virulence factors that can be targets of protective antibody responses (11–13). The flexible set of criteria for M/M-like protein classification has led to ambiguity in the field since various types of M-like proteins have different designations, functions, and sequences (11).

The SzM M-like protein in SEZ has been shown to bind fibrinogen, activate plasminogen, inhibit phagocytosis, and serve as a protective antigen for vaccination (9, 14). However, the contribution of SzM to SEZ virulence *in vivo* has not been determined. SzP, a second SEZ M-like protein, has been shown to contribute to SEZ virulence in an animal model (8, 15). An SzM ortholog in SEE, SeM (also called FgBP [16]), has also been shown to be an important virulence factor when evaluated in mice (16, 17). Dale et al. (18) noted that certain strains of GAS, particularly of the M18 type, encode a SzM ortholog, called Spa (streptococcal protective antigen), which is linked to virulence (19). In the M18 strain studied, a Spa deletion mutant was more attenuated in mice than a mutant with the canonical M18 protein deleted and Spa was a protective antigen as well (19). Although homologs of SzM have been found in several SEZ, SEE, and GAS strains, the distribution and extent of conservation of SzM homologs in strains of these species and in other streptococcal species is unknown. Furthermore, factors governing the expression of SzM or its homologs have not been described. In contrast, the regulation of M protein and two classes of M-like proteins (M-related protein [Mrp] and M-like protein [Enn]) in GAS by the central virulence regulator, Mga (multiple gene activator), is well described (11, 20, 21).

In addition to M/M-like proteins, capsular polysaccharides and other surface polysaccharides are also important streptococcal virulence factors and protective antigens (22–26). For example, the GAS carbohydrate is critical for GAS virulence (22, 23). Poly-*N-*acetyl-d-glucosamine (PNAG) is a surface polysaccharide found on numerous Gram-positive and -negative bacteria and fungi (27, 28) that can serve as a virulence factor and a protective antigen (27, 28). Surface PNAG has been identified in S. pneumoniae, S. pyogenes, and S. dysgalactiae (27), but the loci encoding PNAG biosynthesis have not been described in streptococci. Also, the chemical bases of the association of PNAG with the microbial surface have not been elucidated, and it is unknown whether PNAG is physically linked to the cell membrane or cell wall.

Here, we found that SEZ is bound by a monoclonal antibody to PNAG and performed a fluorescence-activated cell sorting (FACS)-based transposon-insertion sequencing (Tn-seq) screen to identify genes required for surface production of PNAG by SEZ. Unexpectedly, the screen identified SzM and a conserved adjacent locus (termed here SezV for SEZ virulence) that promotes *szM* expression. An anti-PNAG monoclonal antibody was found to directly interact with SzM, suggesting that this M-like protein may be decorated with a PNAG-like glycan. SEZ mutants lacking either *sezV* or *szM* were highly attenuated in a mouse model of infection. Comparative genomics revealed that *szM*, *seM*, and/or *spa* (here referred to as *szM*/*seM*/*spa*) are linked to *sezV* in all SEZ and SEE strains in the database, as well as in all M18 GAS, but not in other streptococci. Thus, our findings suggest that SezV-related regulators and the linked SzM family of M-like proteins define a new subset of virulent streptococci.

## RESULTS

### Tn-seq screen for loci required for surface PNAG expression.

**FIG 1:**
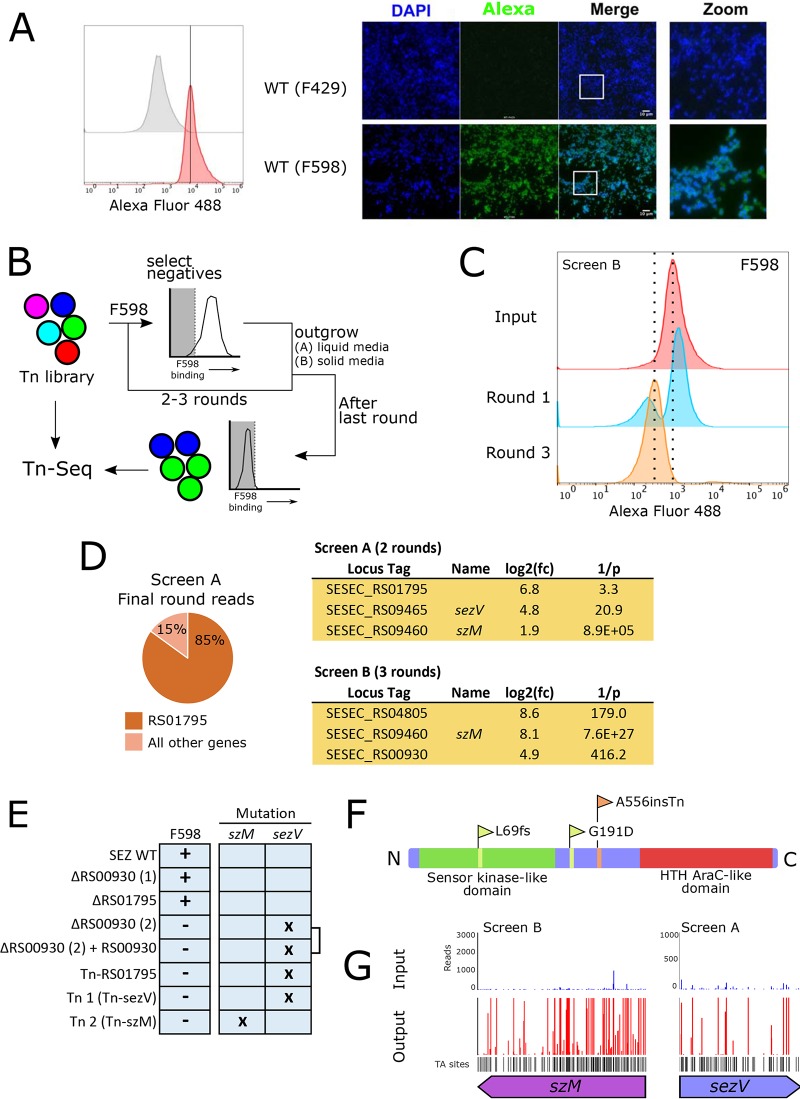
Tn-seq and WGS screens for identification of loci required for surface PNAG expression in SEZ. (A, left) Flow cytometry of SEZ stained with an AF488-conjugated MAb to alginate (F429, negative control) or PNAG (F598). (Right) Microscopy of SEZ stained with DAPI and either AF488-F429 or AF488-F598. The white scale bar represents 10 μm. (B) Schematic for Tn-seq screen for genes required for expression of surface PNAG. (C) AF488-F598 (anti-PNAG) binding to SEZ Tn library at different stages of the Tn-seq screen. Flow cytometry data are taken from screen B. Round 1, after first round of selection; round 3, after third round of selection. Dotted lines are included to help guide the eye. (D, left) Pie chart of the percentage of all reads mapping to a single site in gene RS01795 in the last round of screen A. (Right) Table of selected potential candidate genes required for PNAG expression from Tn-seq screen. (E) AF488-F598 binding phenotype and gene mutated based on WGS of indicated strains. The bracket indicates that strain ΔRS00930 + RS00930 (2), which was derived from strain ΔRS00930 (2), has the exact same mutation in *sezV* as is found in ΔRS00930 (2). Tn1 contained a transposon insertion in *sezV*; Tn2 contained a transposon insertion in *szM*. (F) Location of mutants and domain organization of *sezV*. HTH, helix-turn-helix. Domains in SezV were predicted using HHphred (65, 68) and Phyre2 (69). (G) Comparison of the read count distributions of transposon insertions in *szM* and *sezV* in the input library and in the last round of the screen reveals uniform enrichment of insertions across these genes.

We observed that a porcine SEZ isolate (ATCC 35246) (referred to as SEZ) was bound by a human anti-PNAG monoclonal antibody (MAb F598) (Fig. 1A), and we set out to identify loci that contribute to PNAG synthesis in SEZ. First, we developed a flow cytometry-based system for high-throughput analysis of the PNAG phenotype of single bacteria, using fluorescently conjugated F598 (AF488-F598). There was a considerable range of fluorescence intensities among a population of AF598 bound stationary-phase cells, but this range had minimal overlap with that of cells bound by an AF488-labeled isotype control antibody (F429) (Fig. 1A). Notably, although F429 was raised against an unrelated carbohydrate, it was engineered to include IgG1 heavy- and light-chain constant region amino acid sequences identical to those of F598 (29). Fluorescence microscopy using these two labeled antibodies confirmed that F598, but not F429, bound to SEZ cells (Fig. 1A), consistent with the idea that SEZ expresses surface PNAG.

To generate a genetically heterogeneous population of SEZ containing easily mappable mutations, we developed a method to generate a complex transposon-insertion (Tn) library in SEZ. Using the recently developed pMar4s mariner-based transposon delivery vector (30), a library containing transposons in >50% of potential insertion sites was created (see Fig. S1A and B in the supplemental material). A FACS-based screen of the Tn library with several rounds of selection for bacteria with low fluorescent intensity after staining with AF598 was carried out (Fig. 1B and C). We performed the screen with outgrowth in either liquid (screen A) or solid (screen B) media; more modest gating thresholds were applied in the latter, with the aim of identifying genes with weaker phenotypes. Despite the low (background) fluorescence intensity of all bacterial cells after the final rounds of selection (Fig. 1C), Tn-seq of the selected libraries revealed few genes (screen A, 2; screen B, 16; with average TA sites hit in the input of ≥5, log_2_-fold changes [L2FC] of ≥2, an *P* ≤ 0.05) with statistically significant increased ratios of insertions (fold change values) in the sorted versus the input population (Fig. 1D; see Table S1 in the supplemental material). Notably, after the final round of selection in screen A, there was marked enrichment of only a single Tn insertion (in RS01795; Fig. 1D), suggesting that a jackpot event occurred at some point in the screen. Single gene deletions in two of the top hits (RS01795, WP_014622330.1, L2FC = 6.8, 1/*P* = 3.3; and RS00930, WP_014622184.1, L2FC = 4.9, 1/*P* = 416.2) were created, but neither lost MAb F598 binding (Fig. S1C). A second independently generated ΔRS00930 gene deletion strain lacked MAb F589 binding (Fig. S1C), but complementation with RS00930 did not restore F598 binding, suggesting that RS00930 is not required for F598 binding. The preservation of F598 binding in the single gene deletion mutants suggested that the Tn insertions did not account for the absence of F598 binding in the screen hits.

10.1128/mBio.02500-19.1 FIG S1 Transposon library construction in SEZ and binding of anti-PNAG antibody to mutants identified in Tn-seq screen. (A) Schematic of construction of transposon insertion library in S. equi subspecies *zooepidemicus* strain ATCC 35246. pMar4s (30) is a transposon delivery vector that contains the Himar1 mariner C9 transposase, a transposon with a Kanamycin resistance cassette, and an additional Spectinomycin resistance cassette. pMar4s has a temperature-sensitive origin of replication in SEZ, and will only be propagated at the permissive (cooler) temperature. pMar4s is introduced into WT SEZ via electroporation, and transformants are selected by growing on THY + Spc + Km for 3 days at the permissive temperature of 28°C. 100 colonies are then patched onto THY plates with antibiotics and temperature as indicated to identify colonies in which the transposon vector has not integrated into the genome and verify that there was still a replicating pMar4s plasmid. From the resistance profiles of the 100 colonies, the transposition and integration frequency can be estimated. As indicated, colonies with the desired resistances were picked from a second THY + Spc + Km plate grown at 28°C and frozen stocks were made from these colonies. The transposon library was generating by thawing a frozen stock and spreading it over several large square THY + Km plates that were subsequently grown at 37°C, yielding ∼5.4 × 10^5^ colonies. The colonies were scraped off the plate with THY media, and after adding glycerol were stored as frozen aliquots of the transposon library. (B) Genome and SEZ transposon library statistics. Insertions were identified in 75,610 (52%) of the 146,048 potential Tn insertion sites (i.e., TA dinucleotides). (C) Flow cytometry of indicated strains labeled with AF488-F598. ΔRS00930 (1) and ΔRS00930 (2) are independently derived strains containing deletions of RS00930; ΔRS00930 (2) + RS00930 is the deletion mutant complemented with RS00930. Download FIG S1, EPS file, 0.3 MB. Copyright © 2019 D’Gama et al. 2019 D’Gama et al. This content is distributed under the terms of the Creative Commons Attribution 4.0 International license.

10.1128/mBio.02500-19.8 TABLE S1 Tn-seq data from FACS-based screen and differentially expressed genes in SEZ ATCC 35246 WT versus Δ*sezV* strains found in RNA-seq analysis. Download Table S1, XLSX file, 0.5 MB. Copyright © 2019 D’Gama et al. 2019 D’Gama et al. This content is distributed under the terms of the Creative Commons Attribution 4.0 International license.

Whole-genome sequencing (WGS) was used to identify mutations that were shared by SEZ mutants deficient in F598 binding. Several strains were sequenced, including a few Tn mutants and the RS00930 gene deletion strain that lacked F598 binding. Comparative analyses of these genome sequences revealed that 4 out of 5 F598 negative strains contained one of three distinct mutations in a single gene, RS09465 (WP_038674722.1, old locus tag SeseC_02416), which we have termed *sezV* (for SEZ virulence, Fig. 1E and F). The amino acid sequence of SezV is predicted to contain two domains, an N-terminal sensor kinase-like domain and an AraC helix-turn-helix (HTH) DNA-binding domain, suggesting that the protein is a transcriptional regulator. The remaining F598 negative strain sequenced contained a mutation in the gene coding SzM (RS09460, WP_014623570.1, old locus tag SeseC_02415), a gene adjacent to and divergently transcribed from *sezV* (Fig. 1E and G). None of the three F598-positive strains sequenced contained a mutation in either *sezV* or *szM*. Furthermore, Tn insertions in both *sezV* and *szM* were hits in the Tn-seq screens (Fig. 1D and G), suggesting that these linked loci play roles in presentation of PNAG related epitopes in SEZ and thus facilitate F598 binding.

### Phenotypic characterization of strains lacking *sezV* or *szM*.

**FIG 2:**
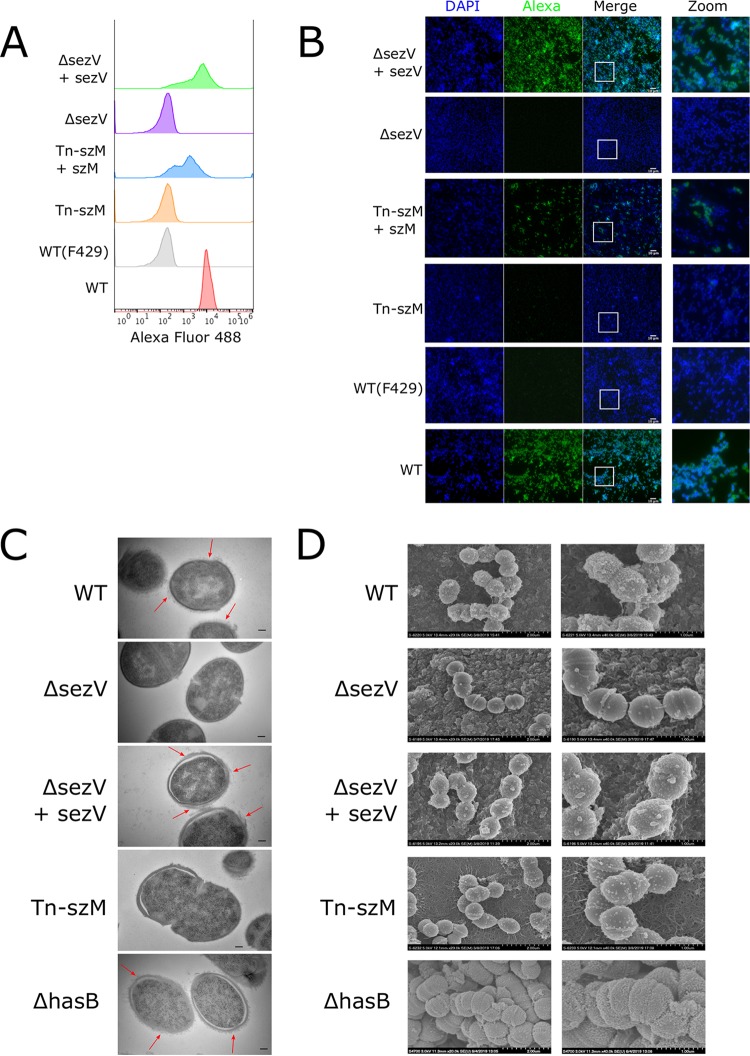
Inactivation of *sezV* or *szM* leads to loss of SEZ reactivity with anti-PNAG antibody and to reduction in surface material. (A) Flow cytometry of indicated strains with AF488-F598. The WT strain was also stained with the negative-control antibody AF488-F429. (B) Microscopy of SEZ strains stained with DAPI and AF488-F598. The WT strain was also stained with AF488-F429. The rightmost panels provide zoomed views of the merged images. The white scale bar represents 10 μm. (C) TEM images of SEZ strains. Red arrows indicate the surface material present on select strains. The black scale bar represents 500 nm. (D) SEM images of the indicated SEZ strains at lower (20K, left) and higher (40K, right) resolutions.

A Δ*sezV* mutant was created, and this strain did not bind AF488-F598 either in flow cytometry or fluorescence microscopy (Fig. 2A and B). Complementation with *sezV* restored the F598 binding activity, establishing that this putative transcription factor is required for binding of this anti-PNAG MAb. We were unable to construct a Δ*szM* mutant, but we found that complementation of the Tn-*szM* mutant (a strain isolated with a transposon insertion in *szM*) with *szM* restored F598 binding activity (Fig. 2A and B).

Transmission electron microscopy (TEM) and scanning electron microscopy (SEM) were used to compare the surfaces of wild-type (WT), Δ*sezV*, and Tn-*szM* mutant cells. In TEM, the Δ*sezV* and Tn-*szM* mutants had smoother outer surfaces than the WT, complemented, and Δ*hasB* strains, which does not produce the SEZ hyaluronic acid capsule (31) (Fig. 2C) . The surface structures in the WT, Δ*sezV* complemented, and Δ*hasB* strains resemble the electron-dense surface “fuzzy coat” attributable to M protein fibers on the surfaces of GAS (32, 33). In SEM, the surfaces of the Δ*sezV* and Tn-*szM* mutants also appeared smoother than the other strains (Fig. 2D). Together, these observations suggest that *sezV* and *szM* are required for the production of a major surface associated component in SEZ.

### *sezV* activates the expression of *szM*.

**FIG 3:**
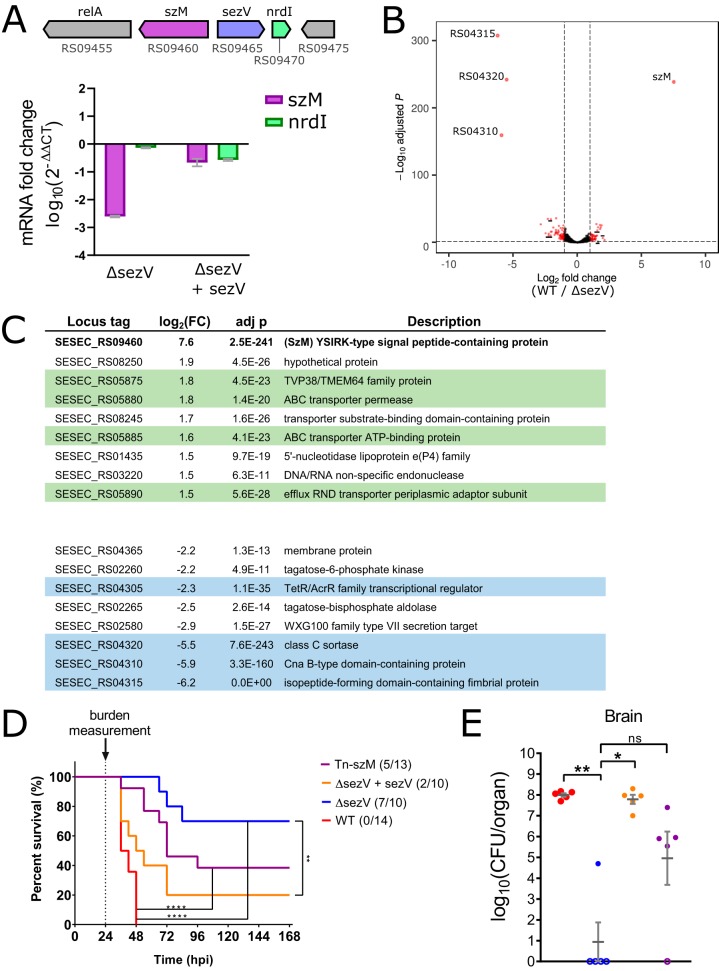
*sezV* is required for expression of *szM* and SEZ virulence. (A, top) Genomic context of *szM* (RS09460) and *sezV* (RS09465) loci in the SEZ ATCC 35246 genome. (Bottom) Relative expression of *szM* and *nrdI* in the Δ*sezV* and Δ*sezV* complemented (Δ*sezV* + *sezV*) strains compared to the WT strain. (B) Volcano plot of RNA-seq results comparing WT and Δ*sezV* strains. The most differentially expressed genes, *szM* and the three genes from the same fimbriae operon (the *cne* homolog-containing Fim1 pilus locus), are labeled. Genes whose expression requires *sezV* have a positive fold change. (C) RNA-seq data for a selected set of genes that includes those with the highest log_2_-fold change [log_2_(FC); WT/Δ*sezV*]. The adjusted *P* value (adj p) from DESeq2 is shown. Genes found in gene clusters or operons are highlighted in the same color. The full list of genes is found in Table S1. (D) Kaplan-Meier survival curves of mice i.v. inoculated with the indicated SEZ strains. Curves were compared using the log-rank (Mantel-Cox) test. Numbers in parentheses refer to animals that died and total animals inoculated (****, *P* < 0.0001; **, *P* < 0.001; *, *P* < 0.05). Comparisons that are nonsignificant are not labeled. (E) SEZ burdens recovered from brains of infected animals at 24 h postinfection. Colors of strains are the same as in panel D. Open circles represent animals for which no CFU were recovered. Groups were compared using a Kruskal-Wallis test with Dunn’s multiple-comparison test (****, *P* < 0.0001; **, *P* < 0.001; *, *P* < 0.05). Comparisons that are nonsignificant are not labeled.

The expression of M/M-like proteins in GAS is activated by *mga*, a gene that is generally found upstream of and transcribed in the same direction as the gene encoding the M/M-like protein. However, no regulator of *szM* (also known as *seM*/*fgbp* or *spa*) has been described. Although *sezV* bears no similarity to *mga*, its predicted domain architecture suggests that it is a transcriptional regulator (Fig. 1F) and the *sezV* gene is adjacent to the *szM* gene in the SEZ genome (Fig. 3A). Quantitative reverse transcription-PCR (RT-PCR) showed that there were reduced levels of *szM* transcripts in the Δ*sezV* background, and this deficiency was complemented by exogenous SezV, strongly suggesting that *sezV* promotes *szM* expression (Fig. 3A). The transcriptomes of the WT and Δ*sezV* strains were compared to identify other loci regulated by *sezV* (Fig. 3B and C; Table S1). This comparison revealed that *sezV* has a relatively small regulon; only one gene, sz*M*, had expression reduced >5-fold in the mutant background, and only three genes, all within a fimbrial operon encoding a Fim1 pilus that includes a *cne* (collagen-binding protein of S. equi) homolog (34, 35), had expression increased >10-fold in the absence of *sezV*. Expression of the gene encoding SzP, the other M-like protein in SEZ, was largely unaffected in the absence of *sezV* (log_2_-fold change of 0.8; WT/Δ*sezV*). Thus, *sezV* appears to primarily activate expression of *szM* and repress expression of a Fim1 pilus locus that encodes a *cne* homolog; notably, Cne has been explored as a candidate component of a vaccine for SEE (36–38).

### *sezV* and *szM* both contribute to SEZ virulence.

A murine model of SEZ virulence (39) was used to test whether *sezV* or *szM* contribute to SEZ pathogenicity. In this model, intravenous (i.v.) inoculation of female adult C57B6/J mice with WT SEZ results in 100% mortality by 48 h postinfection (hpi) (Fig. 3D). The Δ*sezV* strain was highly attenuated and exhibited both delayed kinetics of mortality (0% death at 48 hpi) and diminished absolute lethality (30% dead 1 week after infection). Plasmid-based complementation of the Δ*sezV* mutant largely restored virulence, indicating that *sezV* is a key regulator of SEZ virulence. The Tn-*szM* mutant was also attenuated in this model, revealing that *szM* contributes to SEZ pathogenicity. There was no difference between the Kaplan-Meier curves for the Δ*sezV* and Tn-sz*M* mutants, raising the possibility that the virulence defect of the *sezV* mutant is largely explained by defective *szM* expression in this strain. In addition, the WT, Δ*sezV*, and Tn-*szM* mutant strains grew similarly in culture, suggesting that the attenuation of the mutant strains is not due to growth defects caused by these mutations (Fig. S2A).

10.1128/mBio.02500-19.2 FIG S2 Growth of SEZ strains in culture and *in vivo.* (A) Growth curves of indicated SEZ strains. (B to F) Burden of the indicated strains 24 h after i.v. inoculation. Open circles represent animals for which no CFU were recovered. The results are from the same experiment displayed in Fig. 3E. Download FIG S2, EPS file, 0.4 MB. Copyright © 2019 D’Gama et al. 2019 D’Gama et al. This content is distributed under the terms of the Creative Commons Attribution 4.0 International license.

Given the rapid mortality induced by the WT SEZ strain, organs were collected 24 hpi to measure the bacterial burdens of the four strains in infected host tissues. As observed previously, the highest burden of the WT SEZ strain was found in the brain, reflecting the neurotropism of this pathogen (39). Strikingly, in most mice, no Δ*sezV* CFU were recovered from the brains of infected mice, and this marked virulence defect was corrected by genetic complementation (Fig. 3E). The burden of the Δ*sezV* mutant in the blood and other organs was also reduced compared to the WT strain (Fig. S2B to F), but the magnitude of the defect was not as pronounced as observed in the brain. The burden of the Tn-*szM* mutant in the brain was also reduced, but not as dramatically as the Δ*sezV* mutant; however, the burden of these two mutants was similarly reduced in other organs (Fig. 3E; Fig. S2B to F).

### MAb to PNAG binds SzM.

**FIG 4:**
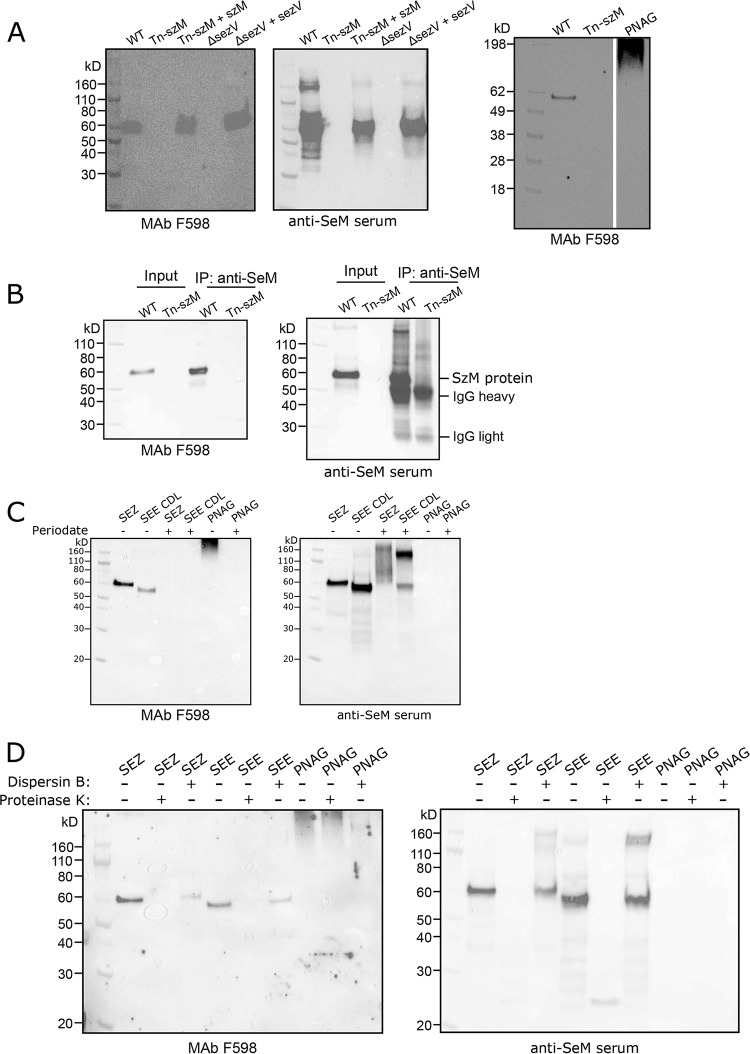
SzM is directly bound by an anti-PNAG antibody. (A) Western blot of bacterial lysates from SEZ strains. Electrophoresed lysates from the indicated strains were run in duplicate on a single SDS-PAGE gel, transferred to a single membrane, which was cut into two parts; each half was blotted with the indicated antibody. (B) Western blot of immunoprecipitated SzM from SEZ WT and Tn-*szM* strains after SDS-Page. (C) Western blot of bacterial lysates of SEZ, SEE strain CDL, or purified PNAG carbohydrate with (+) or without (–) periodate treatment. (D) Western blot of bacterial lysates of SEZ, SEE strain CDL, or purified PNAG carbohydrate with (+) or without (–) treatment with either the dispersin B or proteinase K enzymes.

Our observations that the Tn-*szM* mutant was not recognized by MAb F598 in either flow cytometry or by fluorescence microscopy (Fig. 2) and that F598 binding was restored by expression of *szM* strongly suggest that surface presentation of SzM and PNAG are linked. Immunoblots confirmed this linkage. In Western blots of lysates from WT SEZ, a band of ∼60 kDa, which is slightly greater than the predicted molecular weight of the mature form of SzM (∼57 kDa, after N- and C-terminal processing) was detected with the F598 MAb to PNAG (Fig. 4A). This band was not detected in lysates derived from strains with the transposon insertion in *szM* (Tn-*szM*) or the deletion of *sezV*, but it was restored by complementation of the respective mutations (Fig. 4A). Moreover, a band of apparently the same molecular weight was observed in the same lysates (run on the same SDS-PAGE gel) when antisera raised against the recombinant SEE M-like SeM protein (anti-SeM sera) were used (Fig. 4A). When the blots were probed with MAb F429, an isotype control that was used at the same concentration as F598, bands were not detectable, though they became faintly detectable with longer exposure times (Fig. S3A). Together, these observations suggest the possibility that SzM is decorated by some form of PNAG. However, PNAG is ordinarily an extended capsule-like polymer that barely enters polyacrylamide gels such as the purified material used in Fig. 4A (and Fig. S3B).

10.1128/mBio.02500-19.3 FIG S3 Analysis of SzM binding to isotype control antibody F429, immunoprecipitation of SzM, and glycan mass spectrometry of purified SzM. (A) Western blot of bacterial lysates from SEZ strains after SDS-PAGE with MAb F429. (B) Western blots of bacterial lysates from SEZ strains or purified PNAG carbohydrate after SDS-PAGE with MAb F429. Purified PNAG carbohydrate is not recognized by the isotype control antibody F429. (C) Coomassie blue-stained SDS-PAGE gel of immunoprecipitated SzM. Anti-SeM sera was used to immunoprecipitate SzM from the indicated strains. IgG heavy and light refer to the antibody fragments of the anti-SeM sera. *, An unrelated protein present in WT and Tn-SzM strains that was enriched after immunoprecipitation. (D) Mass spectrometry analysis of polypeptide sequence of SzM from SEZ. After immune- precipitation of SzM, the band corresponding to SzM was cut out and digested with trypsin prior to performing mass spectrometry. Amino acids underlined and in green correspond to regions to which a peptide mapped; no peptide mapped to amino acids in black. We did not expect to obtain coverage of the extreme N and C termini because these are predicted to be cleaved off in the processed, mature form of SzM. The predicted signal sequence [YF]SIRKxxxGxxS[VIA] and cell wall-anchoring motif LPxTG are underlined in black. (E) Glycan mass spectrometry plots of immunoprecipitated SzM (M protein) and control glycoprotein, fetuin (a eukaryotic glycoprotein). Proteins were analyzed for O-glycans (left) and N-glycans (right). No masses corresponding to carbohydrate modifications were found in the O-glycan fraction. In the N-glycan fraction, SzM (M protein) only contained background carbohydrate signals, which were also present in the control sample and do not represent glycosylation of a protein. Expected N-glycans were recovered from the fetuin sample. In both the O- and N-glycan samples of SzM (M protein), no unique carbohydrates were found that were not also found in fetuin. Download FIG S3, EPS file, 1.4 MB. Copyright © 2019 D’Gama et al. 2019 D’Gama et al. This content is distributed under the terms of the Creative Commons Attribution 4.0 International license.

To confirm that the antibody to PNAG and the antibody to M-protein bound to the same protein, SzM was purified using anti-SeM sera; no protein was purified from lysates of Tn-*szM* using the same protocol (Fig. S3C). The purified protein was bound by F598, as well as by anti-SeM (Fig. 4B). Mass spectrometry analysis of the peptides produced by trypsin digestion of the protein immunoprecipitated with the anti-SeM antisera confirmed that it was SzM; peptides were detected that span the majority of the predicted processed form of the protein (i.e., lacking the signal sequence and extreme C terminus, which is presumably cleaved as a result of sortase-mediated attachment of SzM to the cell wall) (Fig. S3D). Together, these observations establish that the MAb F598 to PNAG binds to SzM. F598 also bound to a single band recognized by anti-SeM in lysates of SEE strain CDL (Fig. 4C), suggesting that a PNAG-like molecule may decorate SzM homologs in other species.

To further investigate the chemical bases of the epitopes of SzM and SeM bound by F598, lysates of SEZ and SEE were treated with sodium periodate. This reagent opens the rings between vicinal hydroxyl groups generating aldehydes and is known to disrupt the structure of PNAG such that it is no longer recognized by F598 (27) (Fig. 4C). Periodate-treated bacterial lysates of SEZ and SEE were no longer bound by F598 but retained anti-SeM binding; however, the molecular weights of the proteins recognized by the anti SeM antisera shifted to slower-migrating forms, suggesting that the periodate treatment may have led to the formation of higher-order oligomers of SzM and SeM (Fig. 4C). These lysates were also treated with dispersin B, an enzyme that specifically cleaves the β-1,6 linkage between glucosamines in PNAG (40). After prolonged treatment with this enzyme, there was a marked reduction in F598 binding with only a minor reduction in binding of the anti-SeM antibody (Fig. 4D). Protease treatment ablated reactivity with both F598 and anti-SeM sera but had minimal effect on purified PNAG polysaccharide. Together, these immunochemical experiments on carbohydrate- and protein-specific degradative enzymes’ effects on binding of F598 suggest that SzM and SeM are modified by a carbohydrate with a chemical composition similar to PNAG. Importantly however, we did not detect either O- or N-linked glycans in mass spectrometry analyses of purified SzM protein (Fig. S3E). Thus, we cannot definitively conclude that these M-like proteins are glycosylated.

### Conservation of and variation in *sezV* and *szM/seM/spa*.

**FIG 5:**
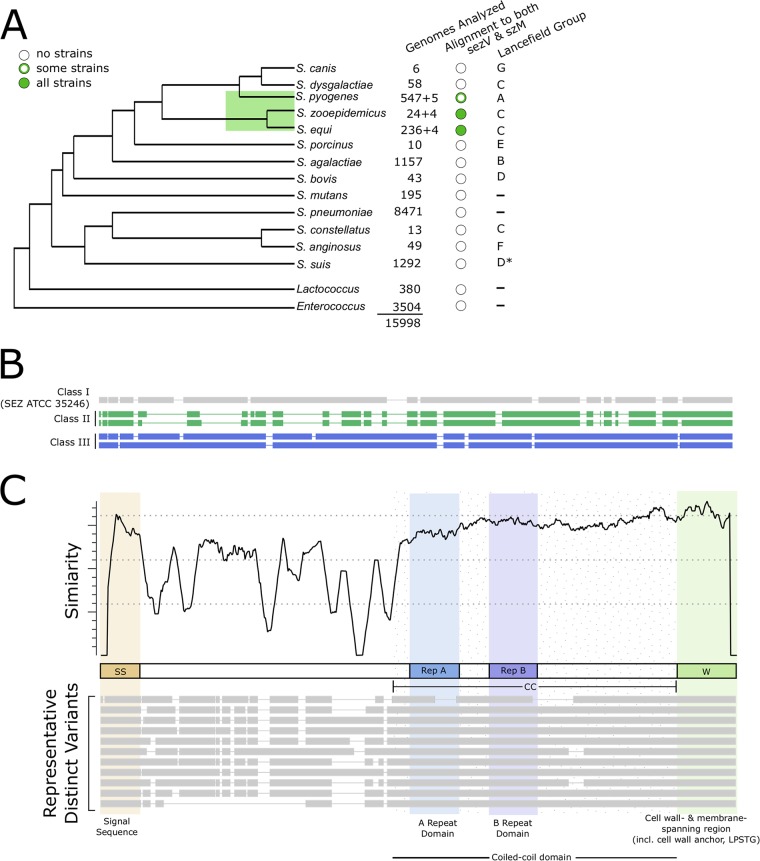
Conservation of *szM/seM/spa* and *sezV*. (A) Conservation of *sezV* and *szM/seM/spa* across various streptococcal species and Lancefield groups and closely related genera. The cladogram is for illustrative purposes and shows a qualitative representation of the relatedness of the species and genera; it was constructed based on the reports of Gao et al. (70) and Hug et al. (71). Branch lengths are not drawn to scale, nor are they a representation of the true genetic distances between species and strains. The genomes analyzed indicate the numbers of genomes queried in each species; +X represents the number of strains newly sequenced in this study. An alignment to *sezV* and *szM* indicates species that have similarity to both genes. Lancefield groups are as reported by Facklam (72) and Okura et al. (73). *, S. suis was formerly classified as group D (73). (B) Three classes of SzM/SeM/Spa-related proteins encoded adjacent to *sezV*. Visualization of multiple sequence alignment between representative members of each of the three protein clusters demonstrates large differences between clusters and similarity within clusters. Lines represent gaps in the multiple sequence alignment; blocks represent continuous sequences. (C) Amino acid sequence variation in SzM/SeM/Spa proteins. (Top) Similarity plot of amino acids at each position in the protein across all variants of the protein. Similarity (*y* axis) represents the relative sequence conservation over a set of adjacent residues; a higher value indicates more conservation. (Middle) Schematic of SzM/SeM/Spa with labeled and shaded domains and regions. SS, signal sequence, which contains YSIRK motif; Rep A/B, repeat A/B; CC, predicted coiled-coil domain; W, cell wall- and cell membrane-spanning region, which contains LPXTG motif. Delineations of regions are based on previously published reports (16, 43, 74); the coiled-coil domain was determined by MARCOIL (75). (Bottom) A multiple sequence alignment of representative SzM/SeM/Spa variants demonstrates various domain/region architectures present across the variants. The lines represent gaps in the multiple sequence alignment; the blocks represent continuous sequences.

Given the importance of *sezV* and *szM* in SEZ virulence, we investigated whether orthologs of these virulence-linked genes exist in publicly available streptococcal genomes. Notably, homologs of *szM/seM/spa* or *sezV*, based on DNA sequence, were restricted to SEZ and SEE in GCS and a subset of GAS strains. We did not find *szM* or *sezV* homologs in any other streptococcal species or Lancefield groups, or in *Lactococcus* or *Enterococcus*, two related genera (Fig. 5A). All strains with *szM* orthologs harbored an adjacent and divergently oriented *sezV* (Fig. S4A and B), suggesting that SezV regulation of SzM is conserved. Among GAS strains, we found that all 15 M18 strains in the database and 1 M36 strain contained the szM ortholog (*spa*) and *sezV* (Fig. S4C).

10.1128/mBio.02500-19.4 FIG S4 Analyses of SzM/SeM/Spa conservation and variants. (A) Distance between start sites of *szM/seM/spa* and *sezV* in strains containing the two genes. In six strains with draft genomes, the two genes were located on different contigs. We excluded these genes from the distance and orientation analyses. (B) Relative orientation of *szM/seM/spa* and *sezV* in strains containing the two genes. The 6 strains in which the two genes were not found on the same contig were excluded from this analysis. (C) GAS strains that contain both *spa* (*szM* ortholog) and *sezV*. (D) N- and C-terminal truncated SzM/SeM/Spa variants discovered in several strains. Translated protein sequences of these genes were not included in additional analyses of *szM/seM/spa* due to the deletion of the signal sequence or cell wall anchoring motif, which will likely render them nonfunctional or not expressed on the cell surface. Species (SEZ, SEE, or GAS) and strain name or WGS identifier is indicated on the left. (E) Distribution of total numbers of strains containing *szM/seM/spa* included in our analyses, and distribution of number of SeM/Spa variants per species. There were a total of 66 SzM/SeM/Spa variants identified. Although the largest percentage of variants belong to SEE, there were ∼10× fewer SEZ strains than SEE strains and the percentage of variants within SEZ is higher than the percentage of SEZ strains in the database analyzed. Hence, the results suggest that SEZ is a more diverse subspecies than SEE. F. Number of SzM/SeM/Spa variants in each species. The diagonal indicates the number of SzM/SeM/Spa variants in each species. The off-diagonal indicates the number of SzM/SeM/spa variants in two species, specified by the row and column header. No variants are shared across a pair of species, or across all three species. Download FIG S4, EPS file, 0.2 MB. Copyright © 2019 D’Gama et al. 2019 D’Gama et al. This content is distributed under the terms of the Creative Commons Attribution 4.0 International license.

The amino acid sequences of SzM/SeM/Spa and SezV from all strains in the database, as well as from 12 newly sequenced strains (see below), were compared. There was considerable variation in the 289 SzM/SeM/Spa amino acid sequences analyzed (Table S2). These M-like sequences could be divided into three classes (Fig. 5B). Most of the sequences (*n* = 274) were similar to SzM from SEZ ATCC 35246; the two other classes, which included seven and eight proteins, respectively, were primarily found in SEZ strains and also encoded adjacent to a *sezV* ortholog. Some strains with truncated SeM/Spa variants were identified (Fig. S4D), which may contribute to the persistence of these strains in their hosts (41), but these were not included in the analyses below.

10.1128/mBio.02500-19.9 TABLE S2 Genomes containing *szM/seM/spa* and *sezV*. Download Table S2, XLSX file, 0.04 MB. Copyright © 2019 D’Gama et al. 2019 D’Gama et al. This content is distributed under the terms of the Creative Commons Attribution 4.0 International license.

There were 66 variants found among the 264 full-length class I SzM/SeM/Spa proteins (Fig. S4E and F). No variants were found in common between species (Fig. S4F). Multiple sequence alignment of the amino acid sequences of the SzM/SeM/Spa variants revealed that most of the variation was found in their respective N termini beginning after their signal sequences (SS) (Fig. 5C), concordant with observations regarding SeM proteins in several SEE strains (42, 43). The highest similarity in the SzM/SeM/Spa proteins was found in their signal sequences, coiled-coil domains, and C-terminal cell wall- and cell membrane-spanning regions (W) (Fig. 5C). The coiled-coil domains contain recognizable A and B repeats, regions of short repeated sequences previously identified in SeM/SzM proteins found in SEE and SEZ (9, 16).

In general, the 289 SezV proteins analyzed exhibited more conservation than the SzM/SeM/Spa proteins (Fig. S5A and Table S2) and have domain organizations and lengths similar to those of SezV from SEZ ATCC 35246. Thus, *sezV* appears to be a conserved transcriptional regulator. However, 39 variants with single amino acid substitutions or small deletions were identified (Fig. S5B), along with several loci that contained more severe changes (Fig. S5C and D).

10.1128/mBio.02500-19.5 FIG S5 Analysis of SezV conservation. (A) Variation in SezV polypeptide sequences. (Top) A similarity plot of amino acids at each position in the protein across all variants. (Bottom) Schematic of SezV with labeled domains. HTH, helix-turn-helix. (B) Distribution of total numbers of strains containing *sezV* included in our analyses, and distribution of number of SezV variants per species. There were a total of 39 SezV variants identified. The greatest number of variants were found among SEZ strains. The percentage of variants belonging to SEZ or GAS was greater than the percentage of the representation of these strains in the database, suggesting that these species have more SezV diversity than found in SEE. (C) N- and C-terminal truncated *sezV* variants identified in several strains. Translated protein sequences of these genes were not included in additional analyses of SezV. Species (SEZ, SEE, or GAS) and strain name or WGS identifier is indicated on the left. (D) *sezV* variants interrupted by an insertion sequence transposase. Download FIG S5, EPS file, 0.1 MB. Copyright © 2019 D’Gama et al. 2019 D’Gama et al. This content is distributed under the terms of the Creative Commons Attribution 4.0 International license.

### SzM/SeM/Spa is recognized by the antibody to PNAG in SEZ, SEE, and M18 GAS strains.

**FIG 6:**
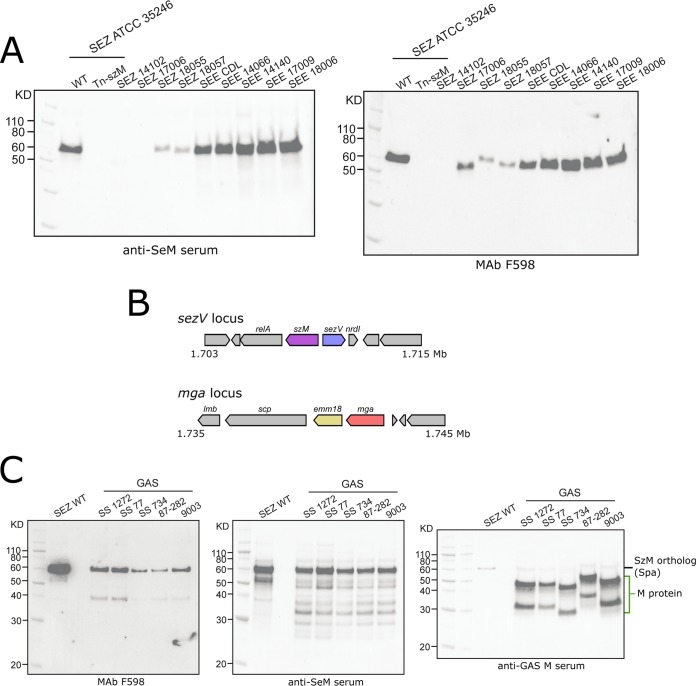
Anti-PNAG antibody binds SzM/SeM/Spa in several SEZ, SEE, and GAS strains. (A) Western blots of bacterial lysates of the indicated SEZ and SEE strains after SDS-PAGE with anti-SeM or F598 antibodies. (B) Genomic context of the *sezV*, *spa* (the *szM* ortholog), and *mga* loci in a canonical M18 GAS strain, MGAS8232 (76). (C) Western blots of bacterial lysates of the indicated SEZ and newly sequenced M18 GAS strains with F598, anti-SeM, or anti-GAS M antibodies. The anti-GAS M serum was sera raised against a purified, canonical GAS M protein. Lower bands in GAS strains may be indicative of M protein degradation products, as seen previously (18).

Given the presence of *szM* and *sezV* orthologs in SEZ, SEE, and M18 GAS strains, we investigated whether MAb F598 to PNAG recognized SzM-related proteins from additional strains. Western blots of bacterial lysates derived from eight SEZ and SEE strains isolated from horses with anti-SeM sera detected proteins of ∼50 to 60 kDa in all strains but longer exposures were needed to detect the reactive band in some SEZ isolates (e.g., SEZ 14102 and 17006, Fig. 6A; Fig. S6A), whose SzM/SeM amino acid sequences differed more from that of SEZ ATCC 35246 (WT) than those from the other SEE isolates. Notably, F598 recognized a protein of size similar to that of the SzM reactive band in all strains tested except for SEZ 14102, an isolate whose SzM sequence contained a 59-amino-acid deletion (Fig. 6A; Fig. S6B). Thus, the anti-PNAG F598 antibody is broadly reactive with diverse SzM/SeM proteins from equine SEZ and SEE isolates.

10.1128/mBio.02500-19.6 FIG S6 SzM/SeM from several SEZ and SEE strains. (A) Western blot of bacterial lysates from SEZ and SEE strains. Longer exposure of the membrane demonstrates expression of SzM in SEZ 14102 and 17006. This is the same membrane shown in Fig. 6A (left). (B) Multiple sequence alignment of SzM/SeM from the indicated strains demonstrates that the 14102 strain has two large deletions in the relatively well conserved C-terminal region of the protein. Download FIG S6, EPS file, 1.5 MB. Copyright © 2019 D’Gama et al. 2019 D’Gama et al. This content is distributed under the terms of the Creative Commons Attribution 4.0 International license.

We also investigated the expression of M and Spa proteins in M18 GAS isolates and whether MAb F598 bound to any of these proteins. The genomes of five clinical M18 isolates were sequenced and assembled (Fig. S7A). All five of these M18 strains contained linked and divergently oriented *sezV* and *szM* orthologs (*spa*). The locus containing *sezV* and *spa* was not linked to the locus encoding the canonical M18 M protein (*emm*) or its associated regulator, *mga* (Fig. 6B). Western blotting of lysates from these five strains with the F598 MAb revealed an ∼60-kDa band, whereas blots with the isotype control MAb F429 were negative (Fig. 6C; Fig. S7B). A very similar-sized band was also detected in blots using anti-SeM serum; this serum also weakly reacted with bands of ∼46 and 34 kDa, which likely correspond to the M18 protein because bands of these sizes were seen in blots with anti-M18 antisera (Fig. 6C). Notably, however, these two bands (likely corresponding to the M18 protein) were not detected with the anti-PNAG antibody. Thus, these M18 GAS strains appear to express at least two M-like proteins, the canonical M18 M protein, which is not recognized by F598, and Spa, which is bound by this anti-PNAG MAb.

10.1128/mBio.02500-19.7 FIG S7 Assembly statistics for newly sequenced GAS strains and lack of binding of the isotype control antibody F429. (A) Assembly statistics for newly sequenced GAS strains. The *emm* type and/or subtype indicated on right was determined via blastp for the M protein and typing via the CDC *emm* blast server. Subtypes are indicated only if the queried M protein had 100% identity over 100% of the subject typing sequence. (B) Western blot of bacterial lysates from SEZ and newly sequenced M18 GAS strains. Download FIG S7, EPS file, 0.7 MB. Copyright © 2019 D’Gama et al. 2019 D’Gama et al. This content is distributed under the terms of the Creative Commons Attribution 4.0 International license.

## DISCUSSION

Our investigation of genes required for surface PNAG reactivity in a porcine SEZ isolate led to the identification of *szM*, which encodes an M-like protein and its linked activator *sezV*. SzM/SeM/Spa (also called FgBP) is representative of a class of M-like proteins distinct from M, Mrp, and Enn. SzM/SeM/Spa proteins appear to be decorated with a PNAG-like oligosaccharide, although this conclusion is based on immunochemical analysis rather than definitive chemical isolation of an oligosaccharide associated with these M-like proteins. Both *szM* and *sezV* are required for robust SEZ virulence, and homologues of these linked virulence genes were identified in all SEZ, SEE, and M18 GAS genomes in the database. Thus, the *szM/seM/spa* and *sezV* loci appear to define a subtype of virulent streptococci.

We used labeled MAb F598 to PNAG to carry out a FACS-based screen of a Tn library in SEZ strain ATCC 35426 to identify insertion mutants that were deficient in surface expression of PNAG. The screen appeared to work well, and even after one round of selection, mutants lacking F598 binding were identified (Fig. 1C); after three rounds of selection, the population was nearly uniformly PNAG negative. However, the library diversity was compromised by Tn jackpots. Linkage between the phenotype (the absence of F598 binding to the Tn insertion mutant) and the genotype (the site of transposon insertion) was not established for several insertions. Whole-genome sequencing led to the identification of mutations in the *sezV* and *szM* loci that were shared among independently derived PNAG-negative SEZ mutants. We were unable to enrich for PNAG-negative cells when a WT (nonmutagenized) SEZ culture was FACS sorted, and it is not clear why PNAG-negative mutants were easily detected in the Tn library.

Although SezV lacks sequence similarity to Mga, it appears to be a functional analogue of this central GAS virulence activator. In GAS, Mga activates expression of virulence-associated and linked M and M-like proteins (Mrp and Enn) (44, 45). Like Mga, SezV proved to be a critical activator of SzM expression and to be required for SEZ virulence in mice. The SezV regulon was relatively small since *szM* was the only locus with a >5-fold reduction in transcript levels in the absence of *sezV*. SezV does not appear to regulate expression of SzP, the other M-like protein in SEZ/SEE, suggesting that there are additional SEZ virulence regulators that have yet to be identified. SezV also appears to downregulate (directly or indirectly) the expression of several genes, including an operon coding for the synthesis of a *cne*-containing fimbrial Fim1 pilus. It is tempting to speculate that the SezV coordinated induction of SzM expression and repression of pilus expression corresponds to a SEZ virulence program that facilitates the organism’s dissemination from sites, such as the oropharynx, where it is a commensal, to sites such as the bloodstream, lungs, and brain, where it is pathogenic. The presence of the predicted sensor kinase-like domain in SezV suggests the possibility that there may be host-derived stimuli that trigger SezV activity.

The key role of SzM in SEZ virulence was not known, but a SzM homologue in SEE (SeM) has been linked to virulence (16, 17). In addition, McLellan et al. (19) found that Spa, a SzM homolog in M18 GAS, appears to a play a more substantial role in pathogenicity than the canonical M18 protein. Similar to canonical M proteins, SzM proteins likely promote virulence through their immunomodulatory functions. Timoney and coworkers have demonstrated that SzM from SEZ strain NC78 can inhibit phagocytosis and bind to and modify the activity of components of the coagulation cascade such as plasminogen (9, 14). In addition to roles in pathogenicity, SzM/SeM/Spa proteins from SEZ, SEE, and M18 GAS strains have proven to be effective immunogens eliciting protective immune responses (9, 14, 16, 18). Our analysis of SzM/SeM/Spa sequences from ∼300 strains revealed that the highest variability is present in the N-terminal regions, whereas the C-terminal coiled-coil domains are more conserved. There appears to be greater variability in SEZ versus SEE, which is consistent with the idea that SEZ as a whole is more genetically variable than SEE (Fig. S4E).

Unexpectedly, SzM and its activator SezV answered the screen for mutants that were not bound by an MAb (F598) to PNAG. Two observations lend support to the idea that SzM is decorated by a PNAG-like carbohydrate. First, purified SzM was bound by F598, whereas F429, an isotype-matched control antibody that includes identical constant regions as F598 bound with much lower affinity. Second, periodate and dispersin B, reagents that cleave carbohydrates, ablated SzM’s reactivity with MAb F598 but not to anti-SeM sera. Specific glycosylation of proteins with oligomers of PNAG (or a related oligosaccharide) has not been described, but O-glycosylation of a streptococcal adhesin has been reported (46). However, it is premature to conclude that SzM is decorated by a PNAG-related oligosaccharide. We did not identify N- or O-linked oligosaccharides bound to SzM by mass spectrometry, and the transposon screen did not yield genes obviously implicated in the synthesis of a polysaccharide. Regardless of whether F598 is recognizing an oligosaccharide linked to SzM, our observation that this antibody binds to a wide range of SEZ, SEE, and M18 GAS strains raises the possibility that immunity to PNAG may have therapeutic applications in animal and human diseases caused by streptococci bearing SzM-like M proteins.

## MATERIALS AND METHODS

### Bacterial strains and growth.

Bacterial strains are listed in Table S3 in the supplemental material. Streptococcal bacteria were routinely grown aerobically in THY (Todd-Hewitt broth plus 0.5% yeast extract) media with shaking or THY agar at 37°C. For GAS strains, liquid cultures were grown aerobically without agitation at 37°C. Antibiotics, when used, were included at the following concentrations: spectinomycin (Spc), 100 μg/ml; or kanamycin (Km), 300 μg/ml. E. coli bacteria were routinely grown in lysogeny broth (LB) media or agar. Antibiotics were used at the following concentrations: carbenicillin (Carb), 100 μg/ml; or Km, 50 μg/ml.

10.1128/mBio.02500-19.10 TABLE S3 Strains, plasmids, and oligonucleotides used in this study. Download Table S3, XLSX file, 0.01 MB. Copyright © 2019 D’Gama et al. 2019 D’Gama et al. This content is distributed under the terms of the Creative Commons Attribution 4.0 International license.

For growth curves, a fresh colony was picked, inoculated in THY media, and grown to log-phase (4 to 6 h) at 37°C. After harvesting by centrifugation, the supernatant was discarded, and each pellet was resuspended in fresh THY to an optical density at 600 nm (OD_600_) of 0.50 and then diluted 10× with the same medium to an OD_600_ of ∼0.05. Then, 200 μl of culture was added per well of a honeycomb plate, with five replicates for each sample. A Bioscreen C (Growth Curves USA) growth curve machine was used to measure the OD_600_ over 24 h, with measurements every 10 min.

### Immunofluorescence microscopy.

Stationary-phase bacteria were labeled with F598 or F429 conjugated to AF488 at room temperature for 4 h after being fixed in ice-cold methanol for 1 min. Widefield microscopy was performed on a Nikon Eclipse Ti microscope. Slides were fixed in ProLong Diamond antifade mountant with DAPI (4′,6′-diamidino-2-phenylindole; Invitrogen).

### Transposon library construction.

A transposon library was constructed in SEZ ATCC 35246 using pMar4s transposon plasmids by modifying a method used for Tn library construction in GAS (30, 47). A detailed graphical depiction of the method can be found in Fig. S1A. Briefly, 1 μg of purified pMar4s plasmid was electroporated into SEZ. Transformants were resuspended in 5 ml of THY plus 10% sucrose recovery broth and then cultured at 28°C for 4 h. Bacteria were spread on a THY plate with Km and Spc after dilution, followed by incubation at 28°C for 3 days. More than 100 individual colonies were screened to verify that pMar4s was present as an independent plasmid (i.e., it had not yet undergone integration or transposition). Each colony was streaked onto six different plates with various antibiotics and grown at 28°C for 3 days or overnight at 37°C, as shown in Fig. S1A. Colonies that were unable to grow on a THY+Spc plate at 37°C were collected from Spc and Km THY plates, and frozen stocks were made (25% glycerol). After reverifying the antibiotic resistance profile of the frozen stock strains, a final Tn library was constructed by spreading >5.4 × 10^5^ colonies from a frozen stock onto 245-cm^2^ square plates (Corning) of THY media, followed by incubation at 37°C overnight. Colonies were scraped from the plate with THY broth and mixed; then, after the addition of glycerol (25% final concentration), the library was frozen at –80°C.

### FACS-based PNAG screen.

Flow cytometry and FACS were performed on a Sony SH800S cell sorter. For routine measurement of antibody binding, live bacteria were labeled with MAb F598 or F429 conjugated to Alexa Fluor 488 (AF488). FACS enrichment of the SEZ transposon library was conducted as follows. An aliquot of the library was stained with AF488-F598 prior to loading on the FACS. From bacteria whose fluorescence intensity was below a specified threshold, the lowest ∼10% (screen A) and ∼20% (screen B) of AF488-F598 labeled bacteria were collected. After enrichment, the bacteria were outgrown overnight either in liquid THY plus 1% dextrose media (A) or on solid THY plates (B) prior to re-enrichment by FACS using a threshold similar to that used for the first round. For bacteria grown on solid THY, prior to FACS sorting, the bacteria were scraped from the plate and resuspended in THY media. With each round, the percentage of bacteria that were below the threshold increased. There were two rounds of selection for screen A and three rounds for screen B. Bacteria from each round and the input were collected, and genomic DNA was extracted for Tn-seq. Several individual bacterial colonies from the final round of each screen were also picked and stored as frozen stocks. Some of these non-F598-binding strains were subjected to WGS, leading to bacterial strains with transposon insertions in RS01795, *sezV* (RS09465), and *szM* (RS09460).

### Tn-seq analysis.

Tn-seq library construction and data analysis was performed as previously described (48–50). Briefly, genomic DNA was extracted, transposon junctions were amplified (a custom primer, Himmer3outMar, was designed for the pMar4s transposon plasmid), sequencing was performed on an Illumina MiSeq, and data were analyzed using a modified ARTIST pipeline. Sequence reads were mapped onto the SEZ ATCC 35246 genome. Reads at each TA site were tallied and assigned to annotated genes. We sequenced the original Tn library, as well as the input Tn library used for the FACS screen (a thawed aliquot of the original Tn library) and the output Tn libraries after FACS selection for absence of F598 binding. For each output sample, a multinomial distribution based on the distribution in the output sample was used to resample reads in the corresponding input sample multiple times and total reads in the simulated inputs was adjusted to match that of the respective output sample. A Mann-Whitney U (MWU) test was used to identify genes that have significantly different insertion profiles and read distributions between the output and simulated input samples. Note that only sites in which there were nonzero reads in either the simulated input and/or output were used, as has been previously described (48). The log_2_-fold change was also calculated for each gene based on the reads in the output and simulated input library. Genes were filtered based on their containing at least five TA sites with a representative mutant in the input library, a log_2_-fold change of at least 1, and an MWU *P* value of at least 0.05.

### Whole-genome sequencing.

Genomic DNA was purified from stationary-phase cultures of bacteria using a MasterPure Gram-positive DNA purification kit (Epicentre, catalog no. MGP04100). The DNA concentration was adjusted prior to library preparation and sequencing at the Biopolymers Facility at Harvard Medical School. Libraries were constructed using the Nextera XT kit (Illumina), and paired-end sequencing was performed on a MiSeq (Illumina) with dual-indexing and 2 × 75-bp reads. Reads were mapped to the SEZ ATCC 35246 reference genome using bwa (51). Nucleotide variants were called as previously described (50), using GATK (v3.5) (52), annotated using ANNOVAR (53), and filtered and compared using vcftools (54). To identify transposon insertions in WGS reads, the reads were processed as for Tn-seq analysis. Putative mutations that result in loss of MAb F598 binding were found by identifying mutations present in the strains that could not bind F598 and absent from strains that could bind F598. For newly sequenced GAS strains, libraries were prepared and sequenced as for SEZ strains. However, in this case, Illumina reads were trimmed with sickle (v1.33) (55) and assembled using SPADES (v3.11.1) (56). Quast (v5.0.2) (57) was used to determine the assembly statistics.

### Strain construction.

Single gene deletions in SEZ ATCC 35246 were constructed as previously described (39) using the temperature-sensitive shuttle plasmid pSET4s (58). Upstream and downstream regions of each gene were cloned into pSET4s, which confers Spc resistance. The constructed plasmids were introduced into competent SEZ cells via electroporation using the following settings: voltage, 2,500 V; capacitance, 25 μF; and resistance, 200 Ω. The transformed bacteria were grown at 37°C in THY broth plus Spc to generate single-crossover mutants. Double-crossover mutants were generated by repeatedly passaging the single-crossover strains at 28°C on THY without Spc. The gene deletions were verified by PCR and Sanger sequencing.

To construct complementation plasmids, the *sezV* and *szM* genes were amplified by PCR from SEZ ATCC 35246 genomic DNA and inserted into the pSET2 plasmid (59). For the *szM* gene, the constructed plasmid was amplified in E. coli DH5α before electroporation into SEZ. For the *sezV* gene, a dialyzed Gibson assembly production of pSET2 and *sezV* was used to directly transform SEZ. For complementation with RS00930, the RS00930 gene, containing a synonymous amino acid mutation, was cloned into pSET4s and used to restore the RS00930 gene in its native chromosomal locus.

PCR was routinely performed using Phusion high-fidelity DNA polymerase (NEB), and plasmids were constructed via Gibson assembly using the NEBuilder HiFi DNA assembly master mix (NEB). Primer sequences are listed in Table S3 in the supplemental material.

### Electron microscopy.

Bacterial samples were submitted to the electron microscopy facility at Harvard Medical School as colonies grown on solid medium THY plates. SEM was performed on bacterial samples fixed via glutaraldehyde.

### Quantitative RT-PCR.

RNA was isolated from 3-ml log-phase cultures of bacteria. After harvesting bacteria via centrifugation, 50 mg of 0.1-mm glass beads were added to the tube, along with 1 ml of TRIzol (Invitrogen). After homogenization of the mixture vigorously for 2 min, chloroform extraction was performed, followed by purification using an RNeasy kit (Qiagen). The RNA was subject to DNase treatment and then reverse transcribed using SuperScript II RT (Invitrogen). Fast SYBR green master mix was used for qPCRs, and all reactions were conducted with an ABI StepOne real-time PCR system. The primers used for qPCR are shown in Table S3.

### RNA sequencing.

RNA was isolated from log-phase cultures of bacteria as described above for qPCR. RNA-seq library construction and sequencing was performed by Genewiz, Inc. Briefly, RNA was depleted of rRNA using an Illumina RiboZero kit, and sequencing libraries were prepared using an NEBNext Ultra kit. Paired-end sequencing of libraries was performed on a single lane of a HiSeq 4000 (Illumina) with single-indexing and 2 × 150 bp reads. The total reads per sample were ∼40+ million, which is well above the number recommended for bacterial differential gene analysis given the genome size of SEZ (60). Reads were mapped to the SEZ ATCC 35246 genome using bwa-mem (51), and read counts per gene were generated using the Rsubread package (v1.34.6) (61) in the R computing environment. Differential gene analysis was performed using the DESeq2 package (v1.24.0) (62) in R. Genes were filtered based on outputs of DESeq2, using a log_2_-fold change magnitude of 1 and an adjusted *P* value of 0.05. A volcano plot was generated using the EnhancedVolcano package in R.

### Animal experiments.

Female C57BL/6J mice (6 to 8 weeks old) obtained from the Jackson Laboratory were used for all experiments. Animal experiments were performed as previously described (39), using protocols approved by the BWH IACUC. Mouse experiments were conducted according to the recommendations of the National Institutes of Health *Guide for the Care and Use of Laboratory Animals*. Adult mice were euthanized via isoflurane inhalation and subsequent cervical dislocation. i.v. infections were performed via tail-vein injection of 10^7^ CFU of log-phase bacteria. To determine tissue burdens, the animals were sacrificed at 24 h after inoculation and dissected, the tissues were homogenized, and serial dilutions of the homogenates were plated on THY media to enumerate bacterial CFU. For the bacterial burden in the blood, prior to sacking, 20 μl of blood was drawn and serial dilutions were plated on THY media.

Data from animal experiments was analyzed in Prism (v8; GraphPad). The Mantel-Cox was used for statistical analyses of the Kaplan-Meier survival curves, and a Kruskal-Wallis test with Dunn's multiple-comparison test was used to compare tissue CFU burdens.

### Western blots.

LDS loading dye (NEB) was added to samples, which were then boiled at 95°C for 10 min prior to running on SDS-PAGE gels. Either Novex Sharp prestained protein standard (Invitrogen) or SeeBlue prestained protein standard (Invitrogen) were used as molecular weight markers. Transfer was performed using the iBlot2 (Thermo Fisher Scientific) system. Membranes were blocked in 1% bovine serum albumin plus 5% powdered milk prior to blotting with primary antibodies. Antibodies were used at the following concentrations: F598 (13 μg/ml), F429 (13 μg/ml), anti-SeM sera (1:1,000), anti-M sera (1:1,000), horseradish peroxidase (HRP)-conjugated goat anti-rabbit IgG antibody (1:10,000, ab6858; Abcam), and HRP-conjugated goat anti-human IgG antibody (1:10,000, A4914; Sigma). The anti-SeM and anti-M sera were both gifts from Gunnar Lindahl. The anti-SeM serum was raised against the full-length SeM protein. The anti-M serum (13) was raised against the C-terminal BCW region, which contains B repeats, the conserved C repeat, and the conserved W (wall-spanning) region of the S. pyogenes M5 protein. SuperSignal chemiluminescent substrates (Thermo Fisher Scientific) were used as the HRP substrates. Gels and Western blots were imaged using the ChemiDoc imaging system (Bio-Rad).

### Immunoprecipitation.

A portion (50 ml) of overnight-cultured bacteria was harvested by centrifugation at 16,000 × *g* for 2 min. After discarding the supernatant and resuspending the pellet in 1 ml of 0.5 M EDTA, the samples were boiled for 5 min to lyse the cells and then centrifuged at ∼20,000 × *g* for 5 min, and the resulting supernatants were used as the bacterial lysates. The lysates were filtered through an Amicon Ultra-4 10K centrifugal filter device to remove EDTA and then mixed with PBS-T (phosphate-buffered saline [pH 7.4] with 0.01% Tween 20). Dynabeads-protein G (Invitrogen) were used for immunoprecipitation. Briefly, 5 μg of F598 or 5 μl of anti-M serum were diluted in 200 μl of PBS-T and rotated for 30 min with 50 μl of magnetic beads at room temperature. The beads were washed once with 200 μl of PBS-T. After removing the supernatant with a magnet rack and adding 1 ml of bacterial lysate with protease inhibitors, the mixture was incubated with rotation for 1 h at room temperature or overnight at 4°C to allow the antigen to bind to the magnetic bead-Ab complex. The beads were washed three times with PBS-T and then eluted by adding 20 μl of 50 mM glycine (pH 2.8), followed by incubation at 70°C for 10 min.

### Periodate and dispersin B treatments.

Bacterial lysates or purified PNAG polysaccharide were mixed with 0.4 M periodate dissolved in PBS at 1:1 (vol/vol), followed by incubation at 37°C for 1 h. The final concentration of dispersin B used for bacterial lysate or PNAG polysaccharide treatment was 500 μg/ml, and samples were incubated at 37°C for 24 h.

### Computational analyses.

The nucleotide sequence of the *sezV-szM* locus from ATCC 35246 plus an additional flanking sequence was used as a query in blastn searches of the NCBI complete and WGS prokaryotic genomes to identify strains of streptococci and related genera that contain the two *szM/seM/spa* and *sezV* genes. Thresholds for identifying homologs were an alignment of >200 nucleotides for *szM* and an alignment of >300 nucleotides for *sezV*. These settings yielded only one homolog in each strain that contained a homolog. SzM homologs were not identified in some strains using more stringent settings; these strains were subsequently identified as containing class II or III *szM* homologs. Protein sequences were aligned using MUSCLE (v3.8.31) (63). Sequence alignments were visualized in Seaview (v4.7) (64) and Genome Workbench (v3.0.0; NCBI).

Protein sequences were clustered with MMSeqs2 (65, 66) using the normal/slow sensitive settings. For identification of representative alleles, 80% minimum sequence identity and 80% minimum alignment coverage was used. For identification of distinct classes of *szM/seM/spa*, the thresholds were both adjusted to 50%. The similarity of amino acids across a multiple sequence alignment was assessed using plotcon (67). Similarity (*y* axis) represents the sequence conservation over a set of adjacent residues. The similarity at one position is calculated as the average of all possible pairwise substitution scores (taken from a similarity matrix) of the residues at the position. The average of position similarities over a set of adjacent residues is graphed in Fig. 5C and Fig. S5A.

Distance between the *szM/seM/spa* and *sezV* genes was determined as the distance between the first base of either gene that occurred first in the strain’s genome and the first base of the second gene. Orientation of transcription of the *szM/seM/spa* and *sezV* genes was determined based on genomic annotations and subsequently compared, taking into account the order in which the genes occurred in the genome.

Identification of the loci in newly sequenced and assembled streptococcal strains was performed using blastn from blast+ (v2.7.1; NCBI). Open reading frames were identified using ORFfinder (NCBI). *emm* (M protein) typing was performed by identifying the annotated M protein in the strain and the CDC *emm* blast server (Streptococcus Group A Subtyping Request Form BLAST 2.0 Server; https://www2a.cdc.gov/ncidod/biotech/strepblast.asp). For newly sequenced GAS strains, the M protein was identified using blast+ (tblastn), the top hit was chosen, and the sequence was then input into the CDC *emm* blast server to type the strain.

M18 strains were identified among the GAS strains using tblastn. The M protein from MGAS 8232 was used as the query sequence to identify additional M18 strains. Thresholds for the E value and the percent identity were lowered until the aligned M protein sequence began to be mapped to a different *emm* type, which was determined manually by inputting the M protein sequences into the CDC *emm* blast server. Based on the final thresholds, a total of 15 M18 strains were identified among the GAS strains analyzed.

### Mass spectrometry.

Mass spectrometry of immunoprecipitated and gel-purified SzM was carried out at the Taplin Biological Mass Spectrometry Facility, Harvard Medical School. In-gel trypsin digestion was performed prior to mass spectrometry analysis. Analysis of O-linked and N-linked glycans on SzM was performed at the National Center for Functional Glycomics. Briefly, N- and O-linked glycans were obtain from SzM and fetuin, a control glycosylated protein, by treating proteins with PNGase F to obtain the N-link glycans and then subsequently with sodium borohydride (NaBH_4_) to obtain the O-linked glycans.

### Data availability.

Illumina reads for the WGS and RNA-seq experiments have been uploaded to the NCBI Short Read Archive (SRA) under BioSample accession numbers SAMN12770224-25, SAMN12772038-42, and SAMN12784884-89.
